# Outcomes of COVID-19 Among Hospitalized Patients With Non-dialysis CKD

**DOI:** 10.3389/fmed.2020.615312

**Published:** 2020-12-03

**Authors:** Armando Coca, Carla Burballa, Francisco Javier Centellas-Pérez, María José Pérez-Sáez, Elena Bustamante-Munguira, Agustín Ortega, Carlos Dueñas, María Dolores Arenas, Juan Pérez-Martínez, Guadalupe Ruiz, Marta Crespo, Francisco Llamas, Juan Bustamante-Munguira, Julio Pascual

**Affiliations:** ^1^Department of Nephrology, Hospital Clínico Universitario Valladolid, Valladolid, Spain; ^2^Grupo de Trabajo de Jóvenes Nefrólogos de la Sociedad Española de Nefrología (JovSEN), Madrid, Spain; ^3^Department of Nephrology, Hospital del Mar Barcelona, Barcelona, Spain; ^4^Department of Nephrology and Renal Transplant, Complejo Hospitalario Universitario de Albacete, Albacete, Spain; ^5^Department of Intensive Care Medicine, Hospital Clínico Universitario Valladolid, Valladolid, Spain; ^6^Department of Internal Medicine, Hospital Clínico Universitario Valladolid, Valladolid, Spain; ^7^Department of Clinical Chemistry, Hospital Clínico Universitario Valladolid, Valladolid, Spain; ^8^Department of Cardiac Surgery, Hospital Clínico Universitario Valladolid, Valladolid, Spain

**Keywords:** chornic kidney disease, pneumonia, COVID−19, acute kidney damage, renal failure

## Abstract

**Background:** Coronavirus disease 2019 (COVID-19), caused by Severe Acute Respiratory Syndrome-Corona Virus 2 has generated significant impact on global health worldwide. COVID-19 can cause pneumonia and organ injury. Chronic kidney disease (CKD) has been associated with increased mortality in previous epidemics, but there is a paucity of data regarding actual risks for non-dialysis CKD patients with COVID-19.

**Methods:** Multicenter, observational cohort study including 136 non-dialysis CKD patients and 136 age- and sex-matched controls that required hospitalization due to COVID-19. Patients with end-stage renal disease, a kidney transplant or without registered baseline glomerular filtration rate prior to COVID-19 infection were excluded. CKD and acute kidney injury (AKI) were defined according to KDIGO criteria.

**Results:** CKD patients had higher white blood cell count and D-dimer and lower lymphocyte percentage. No differences were found regarding symptoms on admission. CKD was associated with higher rate of AKI (61 vs. 24.3%) and mortality (40.4 vs. 24.3%). Patients with AKI had the highest hazard for death (AKI/non-CKD HR:7.04, 95% CI:2.87–17.29; AKI/CKD HR:5.25, 95% CI: 2.29–12.02), followed by CKD subjects without AKI (HR:3.39, 95% CI:1.36–8.46). CKD status did not condition ICU admission or length of in-hospital stay.

**Conclusions:** CKD patients that require hospitalization due to COVID-19 are exposed to higher risk of death and AKI.

## Background

The current pandemic of Coronavirus disease 2019 (COVID-19) caused by Severe Acute Respiratory Syndrome-Corona Virus 2 (SARS-CoV-2) that emerged from China in December 2019 is causing significant impact on global health worldwide (1, 2), affecting more than 24 million people and causing more than 800,000 deaths (as of August 27th, 2020, WHO Coronavirus Disease Dashboard, accessible at https://covid19.who.int). Although in most cases symptoms are mild, COVID-19 can be associated with interstitial and alveolar pneumonia and has the capacity to injure organs such as the heart, nervous system, or the kidney (3, 4).

Reported COVID-19 mortality rates are subject to wide variability, with initial reports from China pointing to fluctuating infection-associated death rates, between 11 and 45% among hospitalized patients (5–7). Age, preexisting conditions such as cardiovascular, cerebrovascular or other underlying diseases, and abnormal inflammatory markers such as low absolute lymphocyte count or elevated D-dimer, C-reactive protein (CRP), or interleukin-6 (IL6) have been associated with increased risk of death (6, 7).

Chronic kidney disease (CKD) is a relevant co-morbidity that has been correlated with increased mortality in previous epidemics, such as influenza, compared to the general population (8). CKD patients tend to be older, suffer more frequently from additional illnesses and experience certain degree of immune dysfunction (8–10). Furthermore, infection is one of the leading causes of mortality in end-stage renal disease (11, 12). CKD patients have shown significantly higher rates of hospitalization for pneumonia and sepsis and lengthier in-hospital stay (13). Infection also occurs three to five times more frequently in patients with early-stage CKD compared to the general population (14). As such, a series of recommendations have been published aiming to mitigate the impact of the pandemic in dialysis and transplant patients (15, 16).

Nevertheless, to the best of our knowledge, there are no published reports on non-dialysis CKD-associated risks during COVID-19. We aimed to analyze the influence of non-dialysis CKD on outcomes in a sample of hospitalized subjects with COVID-19. Secondary objectives included studying incidence and outcomes of superimposed acute kidney injury (AKI) in this setting and the impact of CKD and AKI on the rates of intensive care unit (ICU) admission.

## Methods

### Patient Population and Study Cohort Definitions

We designed a multicenter, observational, age-, and sex-matched cohort study. The study conforms to the STROBE statement for reporting observational studies.

One hundred thirty six adult patients with non-dialysis CKD who required admission for COVID-19 infection due to low oxygen saturation (<90%), dyspnea or pneumonia in three third-level Spanish academic hospitals between March 1st and April, 15th 2020 and 136 age- and sex-matched controls were included in this study. To build the study cohort, all consecutive patients admitted due to COVID-19 infection starting from March 1st, 2020 were chronologically screened. For that purpose, lists provided by hospital admission departments that included patients admitted due to COVID-19 to both the general ward and intensive care units were used, without applying any exclusion criteria. We first built the non-dialysis CKD cohort and searched for age- and sex-matched controls among screened non-CKD patients.

Viral infection was confirmed by real-time RT-qPCR and/or serologic testing in all cases. Disease severity on admission was classified according to the report of the WHO—China Joint Mission on COVID-19 (17). Patients with COVID-19 were divided into mild (laboratory confirmed, without pneumonia), moderate (laboratory confirmed with pneumonia), severe (dyspnea and/or lung infiltrates >50% of the lung field within 24–48 h) and critical (respiratory failure requiring mechanical ventilation, shock, or other organ failure that requires intensive care). Severity criteria based on respiratory frequency, blood oxygen saturation or PaO2/FiO2 ratio were not used due to uneven reporting of such parameters at the time of admission.

The presence of CKD was defined as evidence of sustained estimated glomerular filtration rate (eGFR) <60 and >15 ml/min per 1.73m^2^ within the 6 months prior to COVID-19 hospitalization. Such value had to be confirmed in three or more separated blood tests. When such value was not available (14 patients, 5.14% of the sample), we searched the previous 12 months (18). eGFR was calculated using the Chronic Kidney Disease Epidemiology Collaboration equation (CKD-EPI) (19). Patients requiring maintenance dialysis, kidney transplant recipients and those without registered eGFR values in the 12 months prior to COVID-19 were excluded. The control cohort was 1:1 age- (range +/– 5 years) and sex-matched from the pool of hospitalized COVID-19 patients with baseline eGFR ≥60 ml/min per 1.73m^2^. AKI was defined according to the Kidney Disease: Improving Global Outcomes (KDIGO) guidelines using peak serum creatinine and acute dialysis criteria (20). Briefly:

- AKI stage 1: Serum creatinine increase of 1.5–1.9 times baseline or ≥0.3 mg/dL.
- AKI stage 2: Serum creatinine increase of 2.0–2.9 times baseline.
- AKI stage 3: Serum creatinine increase of ≥3.0 times baseline or ≥4.0 mg/dL or initiation of renal replacement therapy.

Cardiovascular disease was defined as history of myocardial infarction, stroke, or peripheral vascular disease. Chronic lung disease included asthma, chronic obstructive pulmonary disease, and chronic bronchitis. Hematologic disorders included leukemia, lymphoma, plasma cell dyscrasia, myeloproliferative neoplasms, myelofibrosis, and myelodysplastic syndromes.

The study was performed in accordance with the 1975 Declaration of Helsinki. The study protocol was approved by the local ethics committee. Due to its observational nature it was considered exempt from the need to obtain written informed consent from participants (PI 20-1756, Comité de Ética de la Investigación con Medicamentos, Área de Salud Valladolid Este).

### Data Collection

Demographic and clinical data, symptoms, and medical imaging at the time of hospital admission, COVID-19 associated treatment, ICU admission or in-hospital length of stay were gathered from medical records. Information regarding eGFR values prior to COVID-19 hospitalization was obtained in all cases from the electronic laboratory database of each hospital, which included all laboratory tests carried out both in-hospital and in primary care consultations. Primary care and hospital laboratory data were extracted from the patient's charts and electronic laboratory database (Table 1).

**Table 1:** Demographic, clinical characteristics, and COVID-19 therapy among CKD and non-CKD cohorts.

	**All**	**CKD cohort**	**No CKD cohort**	***P*-value**
*N*	272	136	136	
Age, years	80 (70–86)	80 (74–86)	79 (74–86)	0.567
Male sex, *n* (%)	160 (58.8)	80 (58.8)	80 (58.8)	1
Caucasians, *n* (%)	271 (99.6)	136 (100)	135 (99.2)	0.781
Hypertension, n (%)	204 (75)	122 (89.7)	82 (60.2)	<0.001
Diabetes, *n* (%)	86 (31.6)	55 (40.4)	31 (22.8)	0.002
Chronic lung disease, *n* (%)	75 (27.6)	35 (25.7)	40 (29.4)	0.498
Cardiovascular disease, *n* (%)	66 (24.3)	39 (28.7)	27 (19.9)	0.09
Hematologic disorders, *n* (%)	17 (6.3)	11 (8.1)	6 (4.4)	0.21
Cancer, *n* (%)	38 (14)	20 (14.7)	18 (13.2)	0.726
ACEI/ARB use history, *n* (%)	157 (57.7)	96 (70.6)	61 (44.9)	<0.001
Chest radiograph findings upon admission				0.107
Unilateral pneumonia, *n* (%)	73 (26.8)	44 (32.4)	29 (21.3)	
Bilateral pneumonia, *n* (%)	184 (67.6)	86 (63.2)	98 (72.1)	
Non-specific findings/no findings, *n* (%)	15 (5.5)	6 (4.4)	9 (6.6)	
**Symptoms upon admission**				
Fever, *n* (%)	187 (68.8)	94 (69.1)	93 (68.4)	0.896
Dyspnea, *n* (%)	146 (53.7)	73 (53.7)	73 (53.7)	1
Cough, *n* (%)	157 (57.7)	73 (53.7)	84 (61.8)	0.177
Digestive tract symptoms, *n* (%)	86 (31.6)	40 (29.4)	46 (33.8)	0.434
Asymptomatic, *n* (%)	10 (3.7)	4 (2.9)	6 (4.4)	0.519
**Severity**				
Mild, *n* (%)	9 (3.3)	4 (2.9)	5 (3.7)	0.735
Moderate, *n* (%)	49 (18)	33 (24.3)	16 (11.8)	0.007
Severe, *n* (%)	206 (75.7)	94 (69.1)	112 (82.4)	0.011
Critical, *n* (%)	8 (2.9)	5 (3.7)	3 (2.2)	0.473
**Laboratory results upon admission**				
White blood cell count, 10^3^/μl	6.49 (5.14–9.32)	7.69 (5.4–11.18)	6.03 (4.69–8.78)	0.003
Lymphocyte count, 10^3^/μl	0.94 (0.67–1.31)	0.88 (0.65–1.27)	0.98 (0.72–1.33)	0.197
Lymphocyte percentage	14.8 (9.9–22)	12.8 (9–20)	15.8 (11–22.3)	0.028
D-Dimer, ng/ml	944 (598–1,675)	1,089 (670–2,069)	820 (521–1,358)	0.002
C-Reactive protein, mg/l	51 (11–115)	38 (11–134)	63 (11–111)	0.795
Interleukin-6, pg/ml^a^	40 (15.6–67)	43.6 (16.7–83.6)	33.6 (13.1–57.9)	0.098
Baseline serum creatinine, mg/dL	1.02 (0.8–1.42)	1.42 (1.21–1.8)	0.8 (0.68–0.88)	<0.001
Peak serum creatinine, mg/dL	1.26 (0.93–2)	1.97 (1.54–2.99)	0.95 (0.8–1.13)	<0.001
Acute kidney injury				<0.001
No AKI, *n* (%)	156 (57.4)	53 (39)	103 (75.7)	
AKI stage 1, *n* (%)	87 (32)	60 (44.1)	27 (19.9)	
AKI stage 2, *n* (%)	17 (6.3)	14 (10.3)	3 (2.2)	
AKI stage 3, *n* (%)	12 (4.4)	9 (6.6)	3 (2.2)	
**Therapy**				
Lopinavir/ritonavir, *n* (%)	177 (65.1)	87 (64)	90 (66.2)	0.703
Hydroxychloroquine, *n* (%)	253 (93)	122 (89.7)	131 (96.3)	0.032
Antibiotic, *n* (%)	244 (89.7)	122 (89.7)	122 (89.7)	1
Tocilizumab, *n* (%)	5 (1.8)	2 (1.5)	3 (2.2)	0.646
Interferon beta, *n* (%)	80 (29.4)	35 (25.7)	45 (33.1)	0.183

a *Incomplete laboratory data in 8 cases. ACEI, angiotensin-converting-enzyme inhibitors; ARB, angiotensin II receptor blockers; CKD, chronic kidney disease*.

### Study Outcomes

The primary outcome was to describe COVID-19-associated survival during the following 28 days after admission, including eventual deaths after hospital discharge. Secondary outcomes included comparing 28 day incidence of AKI, impact of AKI on outcomes, rates of ICU admission, overall length of stay, laboratory results, and radiological findings caused by COVID-19, as well as administered treatment, in CKD subjects vs. non-CKD patients.

### Statistical Analyses

Demographics were summarized using mean/standard deviations (SD) or median/range for continuous variables and counts/percentages for binary variables, according to data distribution. Continuous data were assessed using *T*-test, Mann–Whitney *U*-test or Kruskal–Wallis test. Binary data were analyzed using the Chi-square test. A two-sided *P* ≤ 0.05 was considered statistically significant. Overall survival probabilities for each cohort were estimated using the Kaplan–Meier method and compared by the log-rank test. *P*-values were adjusted for multiple comparisons using the Benjamini & Hochberg procedure.

We used Cox proportional hazards model with hazard ratios (HRs) with 95% confidence interval (CI), adjusted for age, sex, any comorbidity, angiotensin converting enzyme inhibitor (ACEi)/angiotensin receptor blocker (ARB) use, COVID-19 severity on admission, lymphocyte percentage and C-reactive protein levels on admission, combined CKD/AKI status, and treatment with lopinavir/ritonavir. Comorbidities included hypertension, diabetes, chronic lung disease, cardiovascular disease, hematologic disorders, and cancer. To avoid multicollinearity issues between two highly correlated factors such as CKD and AKI status, a combined variable that included information on both predictors was used. Aalen's additive regression model was calculated to describe the effect of covariates over time as a complement to standard Cox regression. To identify risk factors for ICU admission we used a multivariable competing risk survival model (21) that included all cases with complete laboratory data (264 patients). Subdistribution HR represents as the probability of the event of interest (ICU admission in our analysis) at a specific moment in time considering those who may have died among those still event-free with respect to the event of interest.

Statistical analysis was carried out using the Statistical Package for Social Sciences software, version 20.0 (SPSS, IBM, Armonk, NY, United States), GraphPad Prism, version 7.04 for Windows (GraphPad Software, La Jolla California, United States) and R version 4.0.0 (R Foundation for Statistical Computing, Vienna, Austria) with the survival and cmprsk packages.

## Results

### Baseline Characteristics

A total of 136 patients with CKD and 136 age- and sex-matched controls admitted for COVID-19 were included. All patients were followed until death or the end of the study period. Among the CKD cohort, 53 (38.9%) patients suffered CKD Stage 3a, 54 (39.7%) suffered CKD Stage 3b while 29 (21.3%) presented CKD Stage 4. Due to the matched design of the study, no differences were found regarding age and sex between cohorts. CKD patients suffered hypertension and diabetes and were treated with ACEi and/or ARB drugs more frequently. Prevalence of other comorbidities such as chronic lung disease, cardiovascular, or hematologic disorders was similar between cohorts. We found no differences in reported symptoms or radiograph findings at the time of admission. CKD subjects showed higher white blood cell count and D-dimer levels and lower lymphocyte percentage on admission (Table 1).

### Treatment for COVID-19 and Outcomes

Treatment patterns were similar for both groups except for hydroxychloroquine, which was used less frequently among CKD patients. Both groups showed similar rates of ICU admission and length of stay. However, AKI and mortality rates were significantly higher among CKD patients (Table 2).

**Table 2:** COVID-19 outcomes.

	**All**	**CKD cohort**	**No CKD cohort**	***P*-value**
*N*	272	136	136	
ICU admission, *n* (%)	15 (5.5)	8 (5.9)	7 (5.2)	0.802
Death, *n* (%)	88 (32.4)	55 (40.4)	33 (24.3)	0.004
Length of stay, days	11 (7–18)	11 (7–18)	12 (7–18)	0.739

CKD, chronic kidney disease; ICU, intensive care unit.

### 28-Day Survival

**Figure 1:**
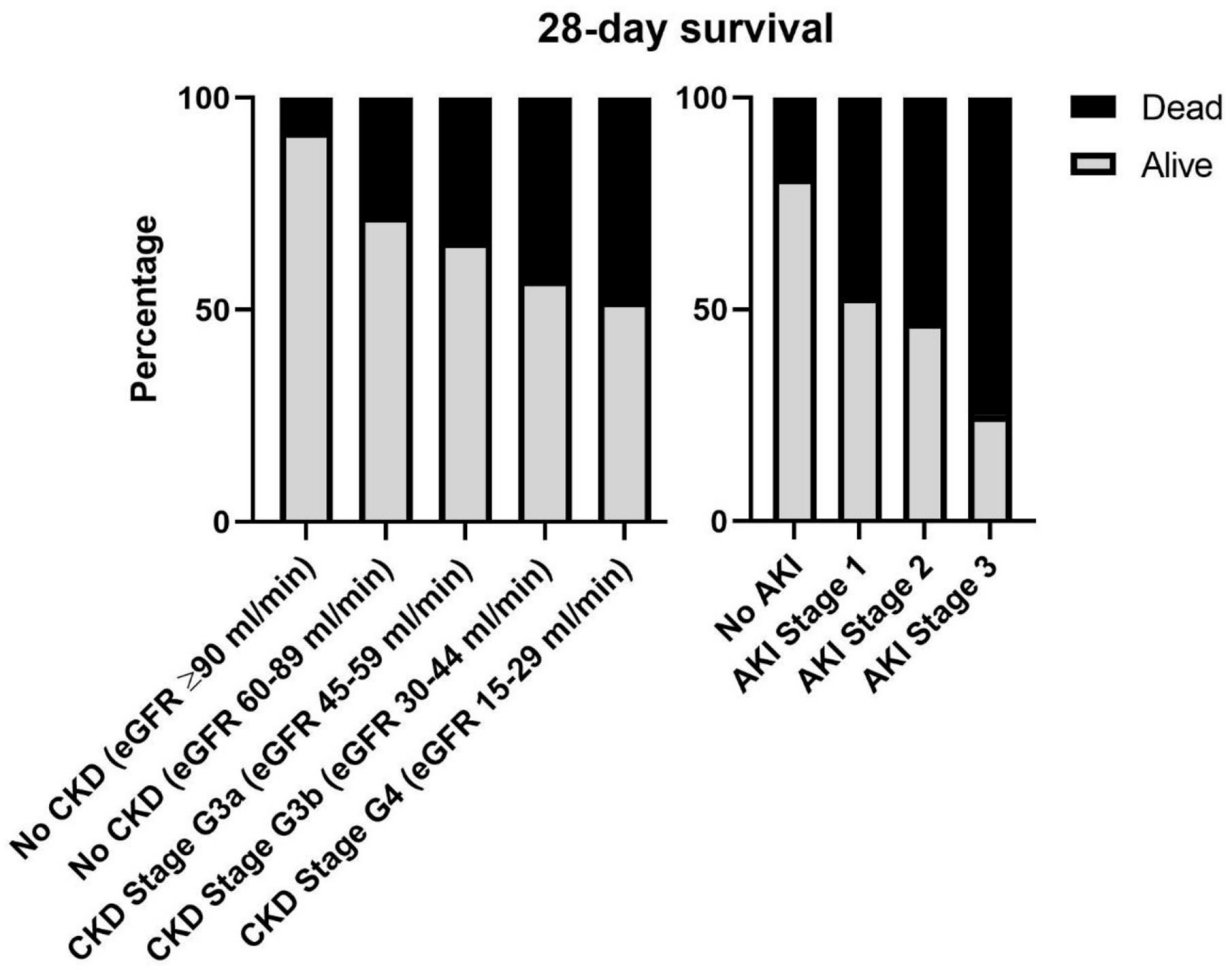
Twenty eight-day survival according to CKD stage and AKI severity. AKI, acute kidney injury; CKD, chronic kidney disease; eGFR, estimated glomerular filtration rate.

**Figure 2:**
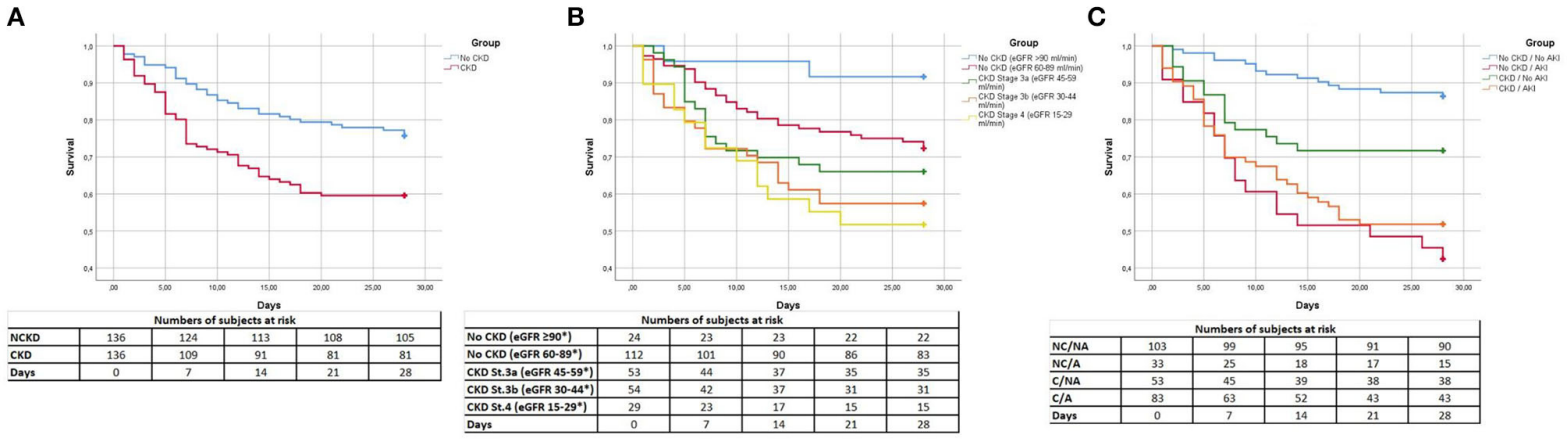
Cumulative Kaplan–Meier survival estimates of the time to patient death. **(A)** Comparison of CKD vs. non-CKD patients. CKD, chronic kidney disease; NCKD, non-chronic kidney disease. **(B)** Comparison of stages of CKD severity. *mL/min/1.73m^2^. St, stage. **(C)** Comparison of CKD and non-CKD patients with and without acute kidney injury. A, acute kidney injury, C, chronic kidney disease, NA, no acute kidney injury. NC, non-chronic kidney disease.

Rates of death increased progressively according to CKD stage and AKI severity (Figure 1). CKD subjects had higher 28-day mortality compared to non-CKD patients (log-Rank: 9.11; *p* = 0.003; Figure 2A). We analyzed survival according to baseline CKD stage. CKD severity was directly associated with worse 28-day survival [log-Rank; Overall:13.99, *p* = 0.007; No CKD (eGFR ≥90 ml/min) vs. No CKD (eGFR 60–89 ml/min):3.71, *p* = 0.09; No CKD (eGFR 60–89 ml/min) vs. CKD Stage 3a:0.98, *p* = 0.372; CKD Stage 3a vs. CKD Stage 3b:0.93, *p* = 0.372; CKD Stage 3b vs. CKD Stage 4:0.22, *p* = 0.642; Figure 2B].

Additionally, we aimed to study the effect of AKI on both cohorts. Subjects with AKI presented the lowest survival independently of CKD status (log-Rank; Overall:37.72, *p* < 0.001; No CKD/No AKI vs. No CKD/AKI:32.97, *p* < 0.001; No CKD/No AKI vs. CKD/No AKI:5.86, *p* = 0.023; No CKD/AKI vs. CKD/No AKI:6.66, *p* = 0.02; No CKD/AKI vs. CKD/AKI:0.65, *p* = 0.422; CKD/No AKI vs. CKD/AKI:4.7, *p* = 0.036; Figure 2C).

**Figure 3:**
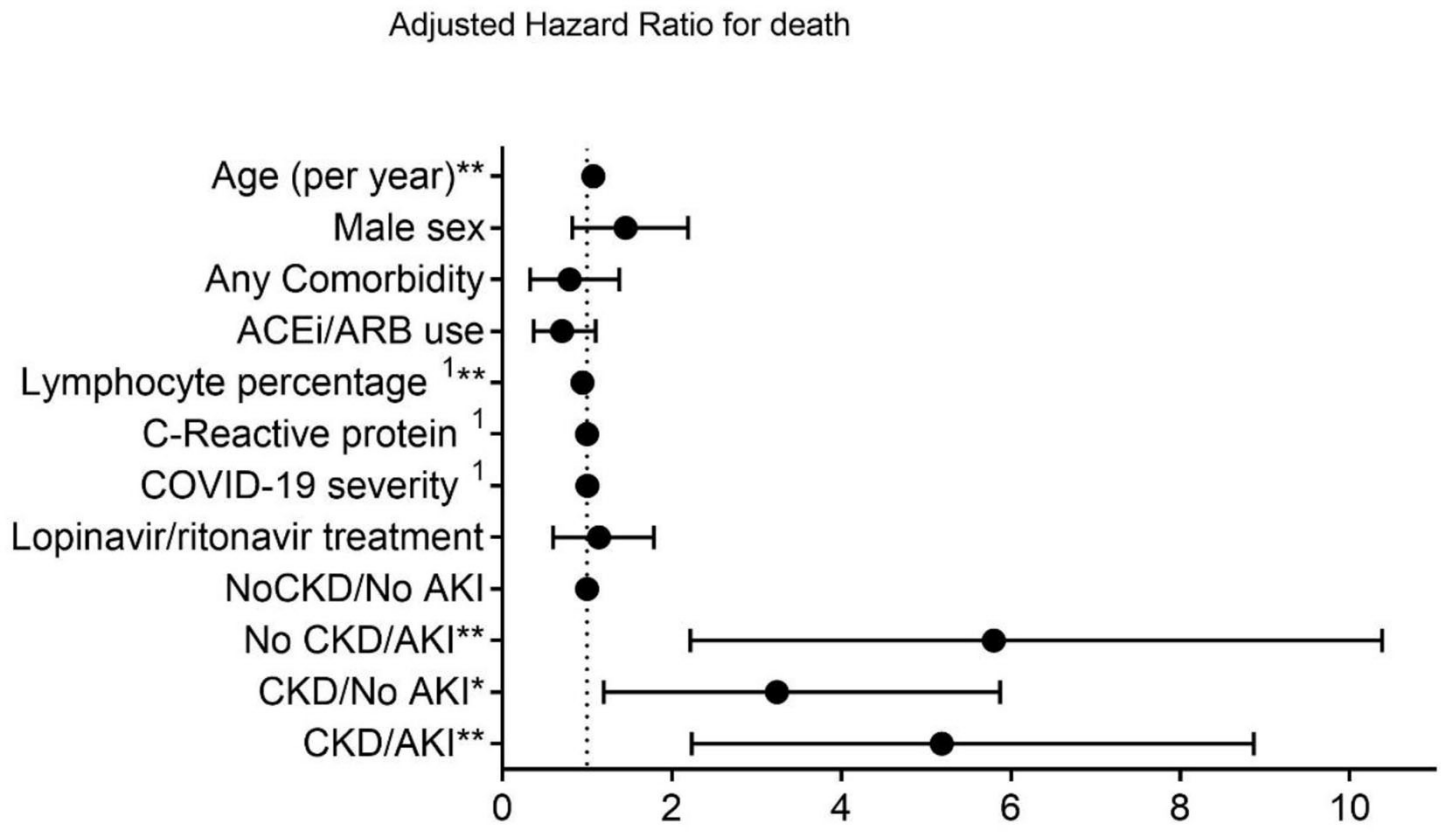
Adjusted proportional hazards model for death. AKI, acute kidney injury; CKD, chronic kidney disease. *p < 0.05, **p < 0.001.

Combined AKI on CKD status was tested as a predictor for 28-day death in Cox regression analysis. Compared with subjects without chronic or acute renal impairment, both non-CKD and CKD patients with AKI had the highest hazard for death (HR:4.8, 95% CI:2.22–10.38, *p* < 0.001 and HR:4.45, 95%CI:2.24–8.87, *p* < 0.001, respectively), in adjusted regression, followed by CKD subjects without AKI (HR:2.65, 95% CI:1.20–5.87, *p* = 0.016). Age and lymphocyte percentage on admission were the only other independent predictors of 28-day mortality (HR:1.08, 95% CI:1.04–1.11, *p* < 0.001 and HR:0.95, 95% CI:0.92–0.98, *p* = 0.001, respectively) (Figure 3).

**Figure 4:**
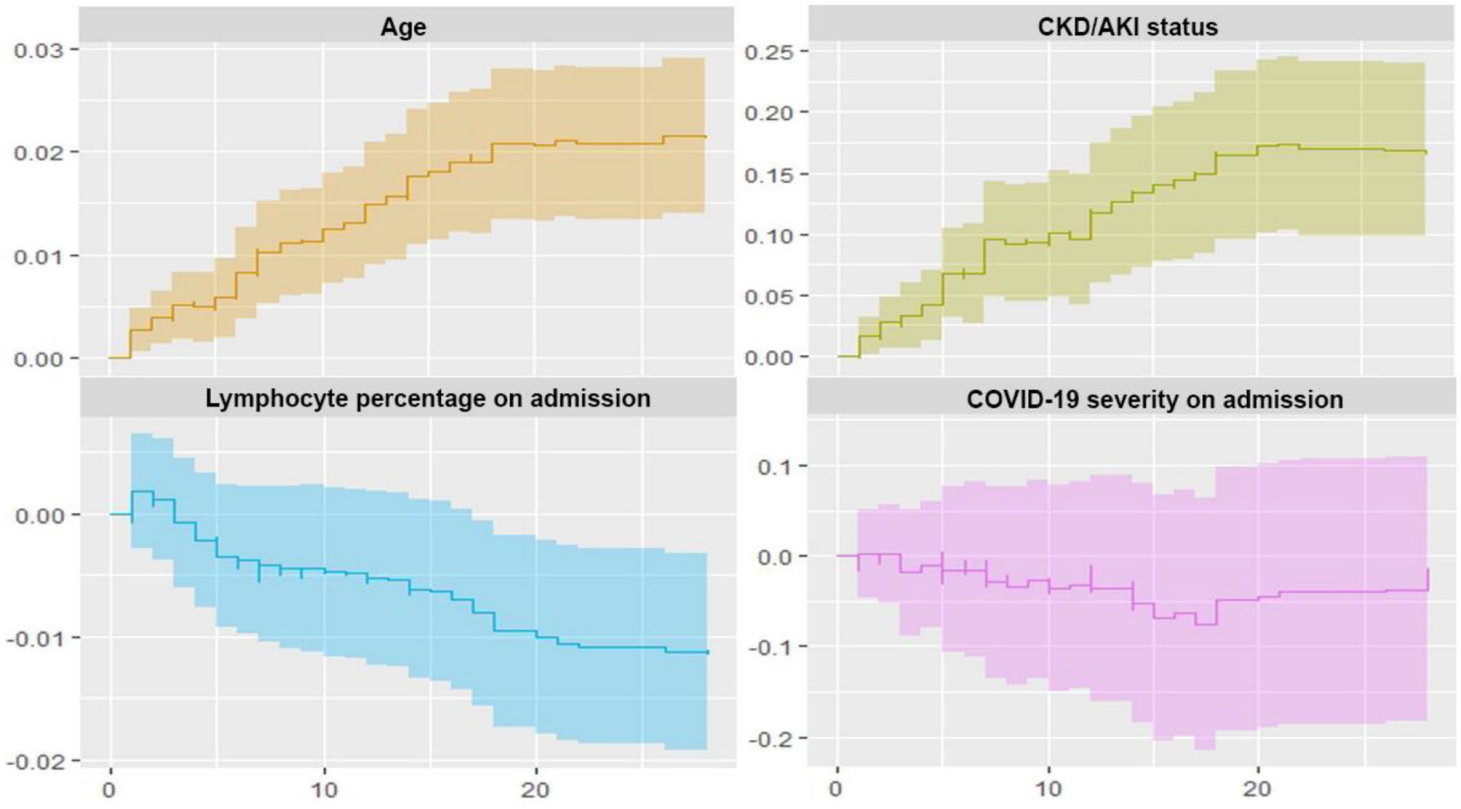
Estimation of cumulative excess risk of based on Aalen's additive model. Aalen's additive model supplements a proportional hazards regression model analysis that describes the nature of time-varying effects of covariates through plots of the estimated cumulative regression coefficients, with confidence bands. AKI, acute kidney injury; CKD, chronic kidney disease.

Figure 4 illustrates cumulative regression functions vs. time with 95% confidence intervals using Aalen's non-parametric additive model. Positive slopes on age and CKD/AKI status suggest an early and maintained increasing effect on the risk for death during approximately the first 20 days after admission. Comparatively, the negative slope on the plot for lymphocyte percentage indicates a continued effect reducing the risk for death during follow-up.

### ICU Admission

Multivariate competing risk survival analysis was used to describe the effect of CKD on ICU admission. CKD status or AKI did not condition ICU admission in these patients. COVID-19 severity and lymphocyte percentage on admission were the only independent predictors of ICU admission in this sample (Table 3).

**Table 3:** Multivariable competing risks analysis for ICU admission.

**Risk factors**	**ICU admission, adjusted cause-specific HR (95% CI)**	**ICU admission, adjusted subdistribution HR (95% CI)**
Age (per year)	0.949 (0.900–1.002)	0.949 (0.892–1.011)
Male sex	0.56 (0.163–1.922)	0.535 (0.143–2.004)
Any comorbidity	0.482 (0.093–2.513)	0.424 (0.059–3.031)
ACEi/ARB use	0.707 (0.204–2.448)	0.791 (0.256–2.44)
CKD	0.675 (0.178–2.569)	0.744 (0.358–1.546)
AKI	2.759 (0.613–12.413)	2.461 (0.588–10.3)
Lymphocyte percentage (per point)^a^	0.819 (0.701–0.958)	0.831 (0.728–0.949)
C-reactive protein (per mg/l)^a^	0.997 (0.991–1.003)	0.996 (0.991–1.001)
COVID-19 severity (per grade)^a^	33.2238 (4.711–234.512)	23.47 (1.374–401.019)
Lopinavir/Ritonavir	8.678 (0.936–80.433)	9.437 (0.869–102.537)

AKI, acute kidney injury; CI, confidence interval; CKD, chronic kidney disease; COVID-19, Coronavirus disease-19; HR, Hazard ratio.

a *On admission*.

## Discussion

Evidence regarding the effect of different chronic conditions on COVID-19 outcomes is scarce. The main finding of our study is that non-dialysis CKD patients suffer from higher risk of 28-day death after admission due to COVID-19 compared to non-CKD subjects. Furthermore, CKD does not appear to significantly modify disease symptoms or radiograph findings on admission, odds of being admitted to the ICU or overall length of stay. Additionally, AKI appears to be a key factor worsening prognosis in both cohorts.

We found that mortality ratios increased progressively as baseline kidney function decreased; subjects with CKD stage 4 exhibited the highest death rate. The association between diminished baseline eGFR and increased risk of infection has already been established, even for low-to-moderate reductions of renal function. Patients with eGFR of 30–59 ml/min suffered a 50% higher risk of infection-related hospitalization, while those with eGFR <30 ml/min presented a risk of death within 30 days of bloodstream infection four times greater than individuals with eGFR >60 ml/min (22, 23). Indeed, in our analysis patients with baseline eGFR between 60 and 90 ml/min presented a death rate three times higher than patients with baseline eGFR >90 ml/min (27.7 vs. 8.3%).

Several pathophysiological mechanisms caused by uremic toxin accumulation, chronic inflammation, increased oxidative stress and endothelial dysfunction are involved in CKD-associated susceptibility to infection (24–27). Chronic decline of renal function is associated with reduced number of B and CD4^+^ T lymphocytes and flawed T-cell response to antigen presentation. The number of naïve circulating CD4^+^ and CD8^+^ T-cells has been linked with serum urea and creatinine levels, which supports our finding that CKD patients had lower total lymphocyte count and lower lymphocyte percentage on admission. Although the number of neutrophils remains stable, they also show higher rate of apoptosis and reduced phagocytic capacity (28–32). T-cell dysfunction is especially relevant as CD8^+^ T-cells appear to regulate the antibody response to SARS coronavirus infection (33). Immune impairment is also conditioned by other non-CKD related factors in these subjects. CKD suffered more frequently from additional comorbidities such as hypertension or diabetes when compared to the general population. The conjunction of general and CKD-specific factors facilitates activation of a proinflammatory phenotype and increased cytotoxicity that interfere with immune system activity.

AKI seems one of the main determinants behind CKD-associated excess mortality in COVID-19 patients. Reported rates for AKI during COVID-19 infection are highly variable. Most published research is based on cohorts from China, with AKI rates ranging from 0.5 to 29%. The first reports in US population described greater rates of AKI, between 19 and 36.6% (5, 34–39). We observed that 42.6% of all subjects developed AKI. This increased incidence rate is probably due to our patients being considerably older and suffering a higher number of comorbidities, especially when compared to Chinese cohorts (2, 36, 37). In addition, CKD subjects suffered AKI more frequently and with increased severity than non-CKD individuals.

CKD has been proven to be a significant risk factor for the development of AKI in patients with hypertension, diabetes, or after major surgery (40, 41). CKD may induce increased propensity to AKI through chronic inflammation, mitochondrial and vascular dysfunction, or abnormal cell signaling and autophagy (42). Increased risk of AKI among patients with elevated baseline serum creatinine has been described in COVID-19 subjects (34). Likewise, COVID-19 can damage kidney tissue, promoting loss of brush border at the proximal tubule, non-isometric vacuolization, and necrosis. Clusters of coronavirus-like particles have been observed in tubular epithelial cells and podocytes, which supports the notion of SARS-CoV-2 renal tropism. Moreover, indirect factors such as systemic hypoxia or coagulation abnormalities commonly associated to COVID-19 pneumonia may further contribute to AKI progression (43, 44).

AKI predicted 28-day mortality in this study, with greater mortality rate in those with the highest severity. Half of the patients who developed AKI died during hospitalization. The combination of AKI and CKD status proved to be the strongest predictor for death in adjusted regression analysis. The link between AKI and death during COVID-19 has been described in Chinese and US cohorts (34, 39, 45) although none of the studies published to date has analyzed the effect of AKI superimposed to CKD. Our results strongly suggest that AKI is key to explain increased COVID-19 mortality in non-dialysis CKD subjects.

In agreement with previous studies (35), age was also an independent risk factor for death in our sample. Chronic comorbidities such as hypertension, diabetes, or cardiovascular disease, which have been linked to increased odds of in-hospital death in COVID-19 patients in previous studies, were not predictors of death during admission. This finding was probably due to the considerably large effect that CKD had over mortality in our sample. The combination of advanced age and high prevalence of CKD in our study may explain the significantly higher mortality registered when compared to previous studies (34, 36). In a meta-analysis performed by Tian et al. that included 14 published observational studies with more than 4,600 patients and only 18 patients with CKD, this disease showed the highest odds ratio for death of all comorbidities analyzed (OR:9.41; 95% CI:3.23–27.4, *p* < 0.0001) (46).

We found no significant differences in symptoms, chest x-ray findings and disease severity on admission in patients with and without CKD. Fever was the most common symptom, followed by cough and dyspnea. Also, bilateral pneumonia was the most common finding on chest x-ray. These findings are in line with previously published data (35, 36). However, CKD patients showed higher levels of D-dimer and a lower lymphocyte percentage, alterations that have been associated with worse COVID-19 prognosis (47, 48). Treatment patterns were also similar in our two cohorts. CKD subjects received less frequently hydroxychloroquine, probably due to potential risk of additional toxicity.

CKD status was not associated with rates of ICU admission or the overall length of hospital stay. In competing risk regression analysis. Only COVID-19 severity and lymphocyte percentage on admission were independent predictors of ICU admission. AKI or other chronic comorbidities were not associated with increased or decreased odds of being admitted to the ICU. Although age failed to predict ICU admission in our study, previous research has shown that COVID-19 patients aged ≥80 years presented a rate of ICU admission of 5.63/100,000, which was considerably lower than that presented by patients with age between 60 and 69 or 70 and 79 (10.29/100,000 and 11.47/100,000, respectively) (49). Potential benefit of ICU admission in the elderly has been subject of debate, as recent research failed to observe a reduction in 6-month mortality or improvement in functional status or quality of life among elderly patients admitted to the ICU vs. standard of care (50).

The present study has limitations. Most patients were elderly Caucasians, limiting extrapolation of results to other groups of different age or ethnicity. Furthermore, only patients with non-dialysis CKD or completely without CKD that required hospitalization were included in the study. Thus, our conclusions should not be projected on patients with milder clinical course. But this study has important strengths. To our knowledge, this is the first study aimed at describing COVID-19 outcomes in non-dialysis CKD patients. We used standardized definitions based on consensus guidelines for CKD and AKI in a multicenter setting. Our sample was built using data from three different academic hospitals including subjects from both the ICU and general ward, increasing the generalization of results to the global hospital setting.

In view of these results we consider that all spectrum of CKD patients is exposed to considerably higher risk of death during hospitalization due to COVID-19. Close monitoring of clinical evolution, lung involvement, inflammatory markers, and renal function and special consideration when administering fluid therapy and/or drugs with potential nephrotoxic effect should be key to avoid complications such as AKI during admission.

In sum, CKD patients that require hospitalization due to COVID-19 suffer from significantly increased risk of death due to higher incidence of AKI during the episode, which considerably worsens prognosis. Further studies should be aimed at better describing the relationship between acute and chronic renal failure in this setting, including medium- and long-term patient and renal outcomes.

## Data Availability Statement

The raw data supporting the conclusions of this article will be made available by the authors, without undue reservation.

## Ethics Statement

The studies involving human participants were reviewed and approved by Comité de Ética de la Investigación con Medicamentos, Área de Salud Valladolid Este. Written informed consent for participation was not required for this study in accordance with the national legislation and the institutional requirements.

## Author Contributions

AC, CB, and FC-P: research design, writing of the paper, and data analysis. AC, CB, FC-P, EB-M, MP-S, AO, CD, MA, JP-M, GR, MC, FL, JB-M, and JP: performance of the research and final approval of the version to be published. All authors contributed to the article and approved the submitted version.

## Conflict of Interest

The authors declare that the research was conducted in the absence of any commercial or financial relationships that could be construed as a potential conflict of interest.
